# A Large Response Range Reflectometric Urea Biosensor Made from Silica-Gel Nanoparticles

**DOI:** 10.3390/s140713186

**Published:** 2014-07-22

**Authors:** Muawia Alqasaimeh, Lee Yook Heng, Musa Ahmad, A.S. Santhana Raj, Tan Ling Ling

**Affiliations:** 1 School of Chemical Sciences and Food Technology, Faculty of Science and Technology, Universiti Kebangsaan Malaysia (UKM), 43600 Bangi, Selangor D.E., Malaysia; E-Mail: mo_qas@yahoo.com; 2 Southeast Asia Disaster Prevention Research Initiative (SEADPRI-UKM), LESTARI, Universiti Kebangsaan Malaysia, 43600 UKM Bangi, Selangor D.E., Malaysia; E-Mail: babybabeoo@gmail.com; 3 Industrial Chemical Technology Programme, Faculty of Science and Technology, Universiti Sains Islam Malaysia, Bandar Baru Nilai, 71800 Nilai, Negeri Sembilan D.K., Malaysia; E-Mail: andong@usim.edu.my; 4 Electron Microscopy Unit, Institute for Medical Research, Jalan Pahang, 50588 Kuala Lumpur, Malaysia; E-Mail: santhana@imr.gov.my

**Keywords:** silica-gel nanospheres, urea biosensor, reflectometric, pH-sensitive, linear response range

## Abstract

A new silica-gel nanospheres (SiO_2_NPs) composition was formulated, followed by biochemical surface functionalization to examine its potential in urea biosensor development. The SiO_2_NPs were basically synthesized based on sol–gel chemistry using a modified Stober method. The SiO_2_NPs surfaces were modified with amine (-NH_2_) functional groups for urease immobilization in the presence of glutaric acid (GA) cross-linker. The chromoionophore pH-sensitive dye ETH 5294 was physically adsorbed on the functionalized SiO_2_NPs as pH transducer. The immobilized urease determined urea concentration reflectometrically based on the colour change of the immobilized chromoionophore as a result of the enzymatic hydrolysis of urea. The pH changes on the biosensor due to the catalytic enzyme reaction of immobilized urease were found to correlate with the urea concentrations over a linear response range of 50–500 mM (R^2^ = 0.96) with a detection limit of 10 mM urea. The biosensor response time was 9 min with reproducibility of less than 10% relative standard deviation (RSD). This optical urea biosensor did not show interferences by Na^+^, K^+^, Mg^2+^ and NH_4_^+^ ions. The biosensor performance has been validated using urine samples in comparison with a non-enzymatic method based on the use of *p*-dimethylaminobenzaldehyde (DMAB) reagent and demonstrated a good correlation between the two different methods (R^2^ = 0.996 and regression slope of 1.0307). The SiO_2_NPs-based reflectometric urea biosensor showed improved dynamic linear response range when compared to other nanoparticle-based optical urea biosensors.

## 1. Introduction

Urea (NH_2_)_2_CO is a nitrogen organic compound commonly found in blood and other bodily fluids. Urea is formed in the kidney from ammonia (NH_3_), which in turn is produced from the breakdown of protein during tissue metabolism. Measurement of urea concentrations in blood provides an indication of kidney and liver functions, congestive heart failure, excessive protein intake or protein catabolism, malnutrition, pregnancy, shock and stress [1–5]. There are many methods known for the determination of urea concentration. Calorimetry, fluorimetry and gas chromatography are conventional methods used for urea concentration determination [5], however, these methods have many disadvantages, e.g., complicated sample pre-treatment, need for skillful operators, high cost of instrumentation and sometimes they require long analysis time [6].

Nanoparticles are interesting materials that bridge the gap between bulk and nano-scale materials. Nanoparticles exhibit electronic, magnetic, ionization potential and optical properties that are different from their bulk counterpart materials. Nanomaterials improve the mechanical properties of both bulk and composite materials [7,8]. To date very few optical urea biosensors have been reported using different kinds of nanoparticles as urease immobilization matrices. Swati *et al.* [9], for instance, reported urease encapsulated in calcium alginate microspheres coated with polyelectrolyte nanofilms containing cresol red dye for optical urea biosensor construction. Duong and Rhee [10] developed luminescent quantum dots incorporated within a sol–gel matrix for urea biosensor construction. Malinowski *et al.* [11] constructed a UV-Vis spectrophotometric urea biosensor based on PANI/cellulose polyacetate membrane for urease immobilization.

**Figure 1.**
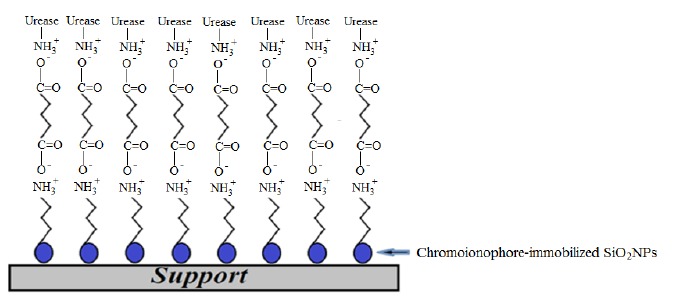
Schematic illustration of the SiO_2_NPs-based optical urea biosensor.

Among all available nanoparticle materials, SiO_2_NPs possess optical, biological and other technologically useful features for industrial applications [12]. They are a non-toxic compound with high chemical stability [13], that can be combined with a diversity of chemical and biochemical surface modifiers to adjust the surface reactivity [14]. In this work, SiO_2_NPs were synthesized based on sol–gel chemistry by hydrolyzing alkyl silicates in a mixed NH_3_/alcohol/water solution [15]. The influence of varying precursor compositions on the SiO_2_NP size distribution was investigated. The optimum composition was then selected for urea biosensor construction. SiO_2_NPs functionalized with -NH_2_ groups and GA cross-linker were used for binding of urease enzyme to the NH_2_-modified SiO_2_NPs (SiO_2_NPs-NH_2_) via ammonium carboxylate salt (-COO^−^ NH_3_^+^-) attachment. A pH-sensitive chromoionophore indicator was physically immobilized onto the SiO_2_NPs. The SiO_2_NPs containing immobilized urease and chromoionophore were finally placed in a plastic case as illustrated in Figure 1.

The immobilized urease catalyzed the hydrolysis of urea and increased the biosensor pH due to the increased number of hydroxyl ions produced (Equation (1)). The resulting increase in alkalinity of the SiO_2_NPs biosensor deprotonated the immobilized chromoionophore and rendered a colour change of chromoionophore from blue to red [16,17], which can be visualized using a reflectance spectrophotometric method:

(1) $${\text{H}}_{2}\text{N}-\text{CO}-{\text{NH}}_{2}+3{\text{H}}_{2}\text{O}\overset{\text{urease}}{\to }2{{\text{NH}}_{4}}^{+}+{{\text{HCO}}_{3}}^{-}+{\text{OH}}^{-}$$

## 2. Materials and Methods

### 2.1. Chemicals

Tetraethylorthosilicate (TEOS, 98%) and 3-aminopropyltriethoxysilane (APS, 99%) were purchased from Aldrich (Louis, MO, USA). Sodium chloride (99.5%), ammonium chloride (99.5%) and urea (99%) from UNIVAR (Seattle, WA, USA). Chromoionophore ETH 5294, magnesium chloride (99.5%), KH_2_PO_4_ (99.5%) and K_2_HPO_4_ were obtained from Fluka (Buchs, Switzerland). NH_3_ solution (25%, A.R.) was purchased from Baker Analyzed (Corporate Parkway, Center Valley, USA), ethanol (EtOH, 95%) from Systerm Company (Zellwood, FL, USA), glutaric acid (GA, 99%) from Merck (Boston, MA, USA), glutaraldehyde (GD, 25% w/w) from Unilap (Doncaster, UK), cetyltrimethylammonium bromide (C_16_TAB, 99%) from BDH-GPR (Lutterworth, UK), potassium chloride (99.5%) from Merck, urease enzyme type III-Jack Bens EC 3.5.1.5 (40,100 units/g) from Sigma-Aldrich and *p*-dimethylaminobenzaldehyde (DMAB, 99%) from Riedel de Haen (Seelze, Germany). Pure water with a resistivity of 18.2 MΩ · cm was used in all solution preparations. All chemicals were used without further purification.

### 2.2. Instrumentation

Reflectance intensity was measured using Ocean Optics Mikropack DH-2000-BAL spectrometer (Dunedin, FL, USA) with a UV-Vis-NIR light source. A personal computer was used for on-line data collection. The instrumental parameters were controlled and data was processed by ocean optic software. A Varian (Cary, NC, USA) Cary 50 UV-Vis spectrophotometer was used for UV-Vis absorption measurements. The surface morphology images of SiO_2_NPs were acquired using scanning electron microscopy (Philips: Peabody, MA, USA). FTIR spectra of SiO_2_ nanoparticle samples were carried out and analyzed by using Perkin Elmer Infrared Spectrometer (Waltham, MA, USA) in the wavenumber range of 400–4000 cm^−1^ with 4 cm^−1^ resolution using KBr disc method.

### 2.3. SiO_2_NPs Preparation and Surface Modification

#### 2.3.1. Synthesis of SiO_2_NPs

SiO_2_NPs were prepared following the conventional Stober method as described in the literature with some modifications [12,15,18–22]. NH_3_ solution (25%) was added to a mixture of ethanol, deionized water, C16TAB and TEOS solution, and vigorously stirred at room temperature (25 °C) for 24 h to obtain a very uniform sized SiO_2_NPs. The resulting SiO_2_NPs were then washed thrice with deionized water via centrifugation followed by decantation and re-suspension in ethanol in an ultrasonic bath. Then, the SiO_2_NPs suspension was centrifuged again at 4000 rpm for 30 min and washed three times with ethanol. Finally, the SiO_2_NPs were dried in a vacuum oven at 60 °C.

#### 2.3.2. SiO_2_NP Surface Modification with -NH_2_ Functional Groups

SiO_2_NP surface modification was carried out based on methods reported in the literature [18,19,21,23,24] with some modifications. The SiO_2_NPs obtained in Section 2.3.1 were treated with an appropriate amount of APS modifier under vigorous stirring to functionalize the SiO_2_NPs with -NH_2_ functional groups. The reaction mixture was kept stirring overnight at ambient temperature. The NH_2_-functionalized SiO_2_NPs were then washed thrice with deionized water via centrifugation followed by decantation and re-suspension in ethanol in an ultrasonic bath. Then, the SiO_2_NPs-NH_2_ suspension was centrifuged again at 4000 rpm for 30 min and washed twice with ethanol. The SiO_2_NPs-NH_2_ was finally dried in a vacuum oven at 50 °C until a constant weight was obtained.

#### 2.3.3. Cross-Linking of SiO_2_NPs-NH_2_ with GD and GA Cross-Linkers

Two types of cross-linkers *i.e.*, GD and GA were used to attach the enzymes to SiO_2_NPs-NH_2_ according to a method reported previously [25–30] with some modifications. Some 16 mL of water was added to 400 mg of SiO_2_NPs-NH_2_ and the mixture sonicated for 5 min until a suspension was obtained. GD (25%, 4 mL) was then added into the suspension and stirred for 24 h. Then, the GD-modified SiO_2_NPs-NH_2_ (SiO_2_NPs-NH_2_–GD) was isolated via 6000 rpm centrifugation for 15 min, washed three times with water and kept until use. The same procedure was applied to modify the SiO_2_NPs-NH_2_ with GA using 1 g of GA with 10 min sonication.

### 2.4. Optical Urea Biosensor Construction

The pH sensor was first prepared prior to the construction of urea biosensor. An adequate amount of pH indicator was added to the GD- or GA-modified SiO_2_NPs-NH_2_ and sonicated until a homogeneous solution was obtained. The mixture was then re-suspended by adding 30 mL water with sonication. The suspended particles were then washed thrice with deionized water followed by centrifugation at 6000 rpm for 15 min. Then, the resulting particles were re-suspended again in 3 mL of 0.05 M phosphate buffer (pH 7) with sonication. Urease enzyme solution (2 mL, 10 mg/mL) was then introduced into the resulting particle suspension and kept at 4 °C for 24 h to allow attachment of urease enzyme onto the surface-modified SiO_2_NPs. The urease-immobilized SiO_2_NPs (urease-SiO_2_NPs) suspension was finally centrifuged at 3000 rpm for 30 min, and washed three times with 0.05 M phosphate buffer (pH 7) to remove the loosely bound urease enzyme. An amount of urease-SiO_2_NPs was collected and placed in an Eppendorf tube cap to be used as an optical urea biosensor.

### 2.5. Optimization of Urea Biosensor

#### 2.5.1. Chromoionophore Concentration Optimization

Different urea biosensors were prepared by immobilizing urease onto GA-modified SiO_2_NPs-NH_2_. The effect of immobilized chromoionophore concentration on the biosensor response was examined from 0.125–0.800 mg chromoionophore/mL ethanol. A calibration curve was obtained for every biosensor in the range of 50–500 mM urea. The biosensor performance was evaluated based on the calculated linear correlation coefficient (R^2^) and sensitivity values

#### 2.5.2. Buffer Capacity and pH Optimizations

Buffer concentration effect was examined between 10 mM and 100 mM phosphate buffer at pH 7 containing 300 mM urea. The biosensor reflectance response was evaluated at the wavelength of 650 nm. For studying the effect of pH, the reaction medium was varied between pH 6 and pH 8 using 50 mM phosphate buffer solution containing 50–500 mM urea. Three calibration curves for urea at pH 6, pH 7 and pH 8 were then plotted.

#### 2.5.3. Evaluation of Urea Biosensor Response

The biosensor response towards different urea concentrations from 50 to 500 mM in 50 mM phosphate buffer (pH 7) was investigated. Maximum reflectance intensity was captured at the wavelength of 650 nm for each urea standard solution with the urea biosensor. Besides, the response time of the urea biosensor was also optimized.

#### 2.5.4. Determination of Urease Loading and Urea Biosensor Stability

Bradford assay is a rapid and sensitive method to determine protein concentration in the solution [31,32]. Bradford reagent was used in the present study to determine the percentage of immobilized urease and the time taken for urease immobilization. A series of urease concentrations in 50 mM phosphate buffer (pH 7) were prepared in the concentration range of 0–4 mg/mL. A 200 μL aliquot from each enzyme solutions were then transferred into separate cuvettes followed by addition of 325 μL deionized water and 1 mL Bradford reagent. The solutions were thoroughly shaken and incubated for five min. Then, the absorbance spectra were recorded within the wavelength range of 595–800 nm. The Bradford standard curve was then constructed based on the absorbance obtained and standard urease concentration. For the determination of unimmobilized urease adhering to the SiO_2_ nanoparticles' surfaces, about 1 mL of urease-SiO_2_NPs from Section 2.4 was centrifuged, and the supernatant liquid was decanted. Some 200 μL of the decanted solution was then mixed with 1 mL of Bradford reagent and 325 μL of deionized water, and incubated for five min followed by absorbance measurement in the wavelength range of 595–800 nm. The incubation time was also varied from 0–90 min to determine the optimum urease incubation time. The stability of the optimized urea biosensor was also assessed. The relative absorbance was calculated and the non-immobilized urease concentration (UI) was estimated based on the Bradford standard curve. And, to determine the amount of immobilized urease, the following equation was used to calculate the percentage of immobilized urease:

(2) %immobilized urease=(4−UI)/4*100

#### 2.5.5. Recovery Study of Urea Biosensor

To evaluate the recovery performance of the urea biosensor, a standard procedure for urea determination based on UV-Vis spectrophotometric and DMAB reagent was adopted [33]. The urea contents in both unspiked and spiked urine samples were determined by both biosensor and non-enzymatic DMAB methods. The DMAB reagent was prepared by dissolving 1.6 g of DMAB in 100 mL ethanol in the presence of 10 mL of concentrated HCl. Urea solutions in the working concentration range of 0.5–7.0 mM were prepared from the urea stock solution. Some 2 mL aliquots of each urea solution was separately pipetted into cuvette and reacted with 2 mL of DMAB reagent. The mixtures were then thoroughly shaken and left for 10 min in a water bath at 25 °C. A blank reagent was also prepared by mixing 2 mL of phosphate buffer solution with 2 mL of DMAB reagent in a cuvette. Absorbance reading was measured at the wavelength of 420 nm. A standard calibration curve for DMAB method was then established and the urea concentrations in urine samples determined by both biosensor and DMAB methods were compared.

#### 2.5.6. Selectivity Study of Urea Biosensor

Na^+^, K^+^, Mg^2+^ and NH_4_^+^ ions' solutions were prepared in the concentration range of 10^−5^–10^−1^ M for interference study of urea biosensor. The urea biosensor was exposed to each interfering ion solution and the reflectance response was obtained at the wavelength of 650 nm. The relationship between reflectance intensity and interfering ion concentration was then plotted. The relative reflectance intensity (ΔInt) was calculated by using Equation (3):

(3) $$\Delta \text{Int}={\text{Int}}_{\text{max}}–{\text{Int}}_{\text{min}}$$

where, Int_max_ represents the maximum reflectance intensity in the presence of interfering ion, and Int_min_ is the minimum reflectance intensity in the presence of the same interfering ion.

## 3. Results and Discussion

### 3.1. Optimization of SiO_2_NPs Composition

**Figure 2.**
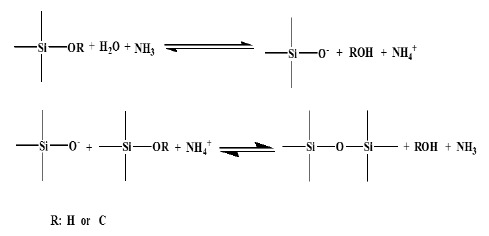
hydrolysis and condensation of TEOS in the Stober method.

In the sol-gel process, three dimensional inorganic networks formation occurred under acidic or basic medium for the colloidal suspension forming nanometer particles. Three reactions occur in the sol-gel process, namely hydrolysis of an alkoxide, alcohol condensation and water condensation, which then is converted into a gel through polycondensation of the sol [34–37]. The hydrolysis and condensation of TEOS in the Stober method can be illustrated in Figure 2 [38]:

A supersaturated solution of silicic acid occurred as a result of TEOS hydrolysis and condensation of silicic acid as small primary SiO_2_NPs with diameters of less than 5 nm. The unstable primary particle aggregates are ready to aggregate into bigger particles with large surface charge to prevent the irreversible Brownian aggregation. The SiO_2_NPs could aggregate with each other due to the chemical affinity of their surfaces to SiO_2_. Thus, further deposition of silica may allow the formation and assembly into the secondary submicron-sized bigger particles [13,38].

As described in the literature, the size of SiO_2_NP correlates with the molar ratio of NH_3_, alcohol, TEOS and water. The nanoparticles size depends on two factors: (1) the reaction kinetics and (2) the colloidal stability, which depends on thermodynamic effects [39]. Surfactant (e.g., C16TAB) is another reported factor could affect the size and distribution of SiO_2_NPs synthesis [40–43].

In this work, an investigation between SiO_2_NPs size and its chemical composition was studied at 25 °C. The SiO_2_NPs chemical compositions prepared under basic medium and the average particle sizes obtained is tabulated in Table 1.

**Figure 3.**
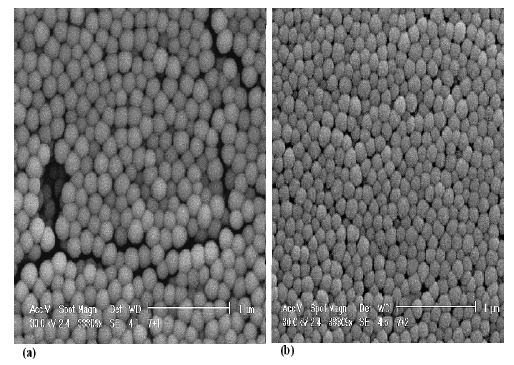
SEM images of SiO_2_NPs for (**a**) t1 and (**b**) t2 compositions.

It was found that the higher the volume of ethanol or water the smaller the SiO_2_NPs size obtained, while higher TEOS or NH_3_ amount produced larger SiO_2_NPs size. t2 denotes the optimized SiO_2_NPs precursor composition with the smallest SiO_2_NPs size obtained at 153.94 nm. The size distributions of SiO_2_NPs for t1 and t2 compositions are shown in Figure 3.

### 3.2. Biochemically Functionalized SiO_2_NPs

**Figure 4.**
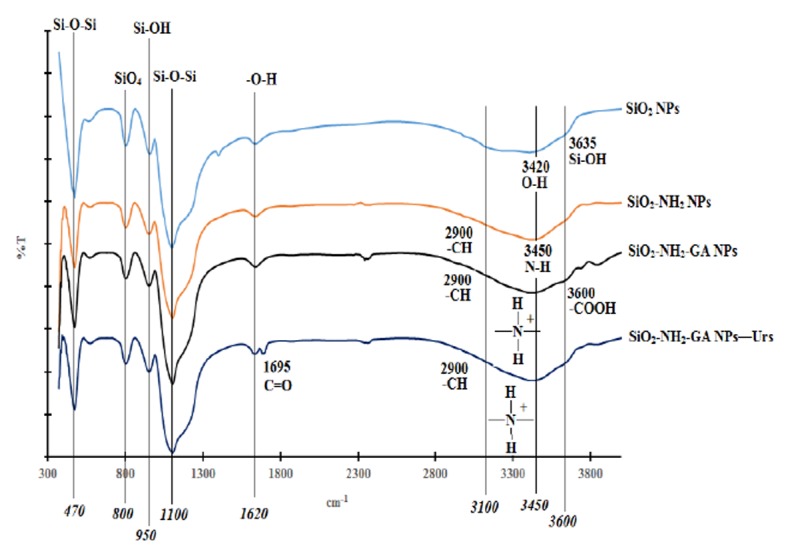
The FTIR spectra for SiO_2_NPs (t2 composition), SiO_2_NPs-NH_2_, SiO_2_NPs-NH_2_-GA and SiO_2_NPs-NH_2_-GA-urease.

APS was used as a chemical modifier to modify the SiO_2_NPs with -NH_2_ groups. GA and GD were used to bind the urease enzyme to SiO_2_NPs-NH_2_.The white coloured SiO_2_NPs-NH_2_ was noticed to turn to a red colour when cross-linked to GD cross-linker, and the particles remained white in colour with GA cross-linking agent. The white coloured GA-cross-linked SiO_2_NPs-NH_2_ was more useful for pH dye indicator immobilization, therefore it was chosen for subsequent urea biosensor construction. Figure 4 illustrates the FTIR spectra of SiO_2_NPs prepared from the t2 composition, SiO_2_NPs-NH_2_, SiO_2_NPs-NH_2_-GA and SiO_2_NPs-NH_2_-GA-urease.

The pure SiO_2_NPs has two strong FTIR absorption bands at ∼1100 cm^−1^ and ∼470 cm^−1^ due to the extension and flexural vibrations of Si-O-Si bonds. The FTIR absorption band at ∼800 cm^−1^ corresponds to the vibrations of SiO_4_ tetrahedrons. The absorption bands at ∼3420 and ∼1620 cm^−1^ are attributed to the extension and flexural vibrations of O-H bonds in adsorbed water. The absorption bands at ∼3635 and ∼954 cm^−1^ are due to the vibration of Si-OH bonds [20,44–46].

Absorption bands at ∼470 cm^−1^, ∼800 cm^−1^, ∼950 cm^−1^, ∼1100 cm^−1^ and ∼1620 cm^−1^ are observed in SiO_2_NPs-NH_2_, SiO_2_NPs-NH_2_-GA and SiO_2_NPs-NH_2_-GA-urease. These absorption bands are consistent with the previously reported pure SiO_2_NPs [47]. The FTIR spectrum of SiO_2_NPs-NH_2_ shows a broad FTIR absorption band from approximately 3100 to 3700 cm^−1^, which correspond to hydrogen bonds and partially hydrated silanols. The spectrum shows an FTIR absorption band around 3100 cm^−1^ from the N-H stretching mode as well as some weaker bands around 2900 cm^−1^ from the –CH and –CH– stretching modes of alkylamine chains [18,47].

**Figure 5.**
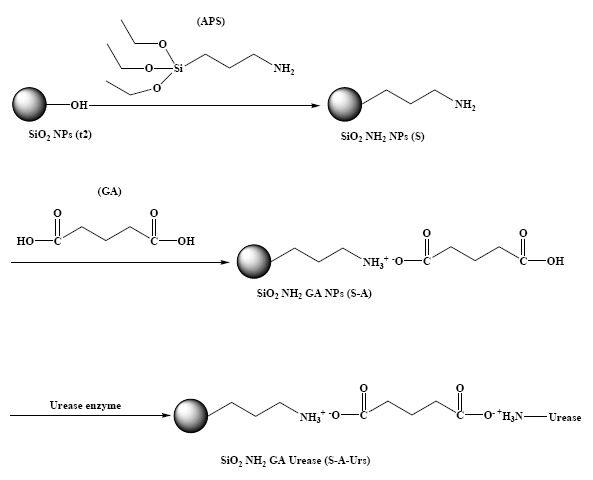
Schematic illustration for SiO_2_NPs biochemical surface modification.

The peak corresponding to N-H stretching is overlapped with the O–H stretching vibrations at ∼3450 cm^−1^ [47]. Also, C-H stretching bands are seen at ∼3000 cm^−1^ confirming the presence of propyl chains with -NH_2_ functional groups [18]. The FTIR spectrum for SiO_2_NPs-NH_2_-GA shows simultaneous appearance of a shoulder at about 3600 cm^−1^, which corresponds to the O-H stretching mode of -COOH functional groups [18]. The strong broad peak observed in the 3100–3450 cm^−1^ region is attributed to the presence of ammonium (NH_4_^+^) ions [48]. The FTIR spectrum for SiO_2_ NPs-NH_2_-GA-urease shows an additional peak at 1695 cm^−1^ that corresponds to C=O stretching related to the amide I band of urease which indicates the presence of immobilized Urs (Ahuja *et al.* [49]). The FTIR spectrum for SiO_2_NPs-NH_2_-GA-urease shows the disappearance of a shoulder at absorption band of 3600 cm^−1^ due to the reaction between the -COOH group of immobilized GA and the -NH_2_ group of the enzyme forming an amide (-CONH) bond. Based on FTIR structural analysis, Figure 5 shows a schematic illustration for the SiO_2_NPs' biochemical surface modification.

**Figure 6.**
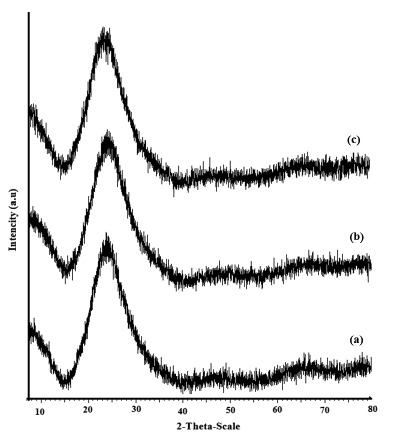
The XRD spectra for (**a**) SiO_2_NPs (t2 composition); (**b**) SiO_2_NPs-NH_2_ and (**c**) SiO_2_NPs-NH_2_-GA.

Figure 6 shows the XRD spectra for SiO_2_NPs (t2 composition), SiO_2_NPs-NH_2_ and SiO_2_NPs-NH_2_-GA. At 2θ ≈ 24, broad peaks are observed for each spectrum, which are assigned to amorphous structures of silica gel nanoparticles, and indicate the small size or incomplete inner structures of these particles. This demonstrated that a high percentage of these particles are amorphous [50–55].

### 3.3. Optimization Studies of Urea Biosensor Response

#### 3.3.1. Optimization of Chromoionophore Concentration

Chromoionophore ETH 5294 has an excellent selectivity to proton donors with a wide range of p*K*a values. The chromoionophore demonstrates changes in optical properties during protonation and deprotonation reactions. It is often used as a pH sensitive dye that forms a blue coloration during protonated state and a red coloration in deprotonated state [16,17]. It has optical transduction properties, which can be used in conjunction with other sensing reagents to detect a wide variety of analytes [56]. When urea is hydrolyzed by the immobilized urease enzyme, the higher hydroxyl ion concentration causes an increase in pH of the reaction medium, and this leads to a colour change in the immobilized chromoionophore, which can be related to the urea concentration [57].

Reflectance spectroscopy investigates the spectral composition of surface-reflected optical radiation, which referred to its angularly dependent intensity and composition of the incident radiation [58,59]. The blue coloured immobilized chromoionophore gives a darker background to the biosensor, which reduces the optical reflected radiation intensity. In view of the variation in reflected light intensity corresponds to the immobilized chromoinophore concentration, an optimization on the immobilized chromoionophore concentration was conducted.

Some four biosensors were prepared with different chromoionophore concentrations. Each biosensor was analyzed within a urea concentration range of 50–500 mM at maximum reflectance wavelengths of 650 nm (for biosensor B1, B2 and B3) and 760 nm (for biosensor B4).The trends of the relation between reflectance intensity and urea concentration with different chromoionophore loadings were plotted. The R^2^ and sensitivity values of each response curve were calculated and are listed in Table 2. Biosensor B2 showed optimum response with good sensitivity and satisfactory R^2^ values compared to biosensor B1, B3 and B4. Thus, biosensor B2 was used for further optimization.

#### 3.3.2. Optimization of Urease Immobilization Duration

**Figure 7.**
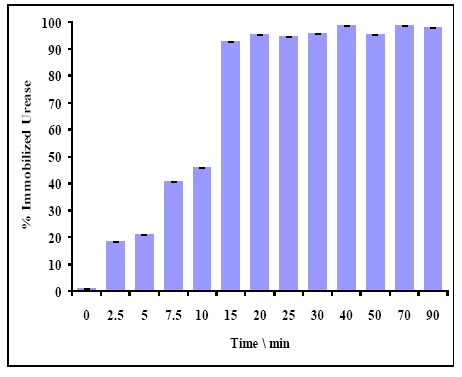
Urease incubation period *versus* percentage of immobilized urease (*n* = 3).

Figure 7 shows the amount of immobilized urease with different enzyme incubation durations. It was noticed that 15 min of enzyme incubation period was sufficient to obtain 100% immobilized urease.

#### 3.3.3. Effect of Buffer Concentration on Biosensor Response

**Figure 8.**
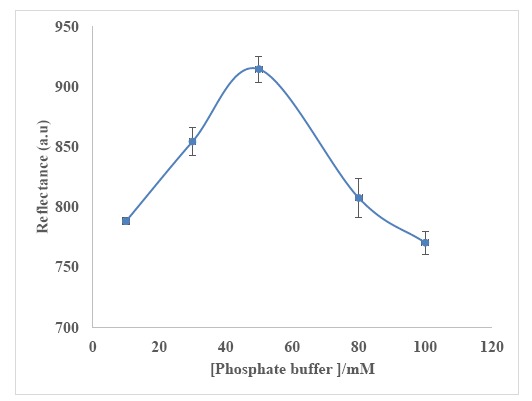
Effect of buffer concentration on urea biosensor response at 650 nm using 300 mM urea at pH 7 (*n* = 3).

Buffer capacity is an important parameter that must be taken into account to compromise between a high response, repeatability and sensitivity to fluctuations [60,61] of the urea biosensor. Three biosensors were analyzed in the same phosphate buffer concentration range from 10 to 100 mM (pH 7) containing 300 mM urea. The maximum reflectance intensity at 650 nm was obtained at 50 mM phosphate buffer (pH 7) (Figure 8), and this buffer concentration was used throughout the biosensor optimization experiments. The biosensor response declined at phosphate buffer concentration higher than 50 mM due to quenching of enzymatic reaction, whilst lower phosphate buffer concentration gave low ionic concentration effect [60,61].

#### 3.3.4. Optimization of Buffer pH

The buffer pH has an effect on urease hydrolysis rate [62]. Basically, the variation in pH or temperature medium has an ionic strength impact on the enzyme due to changes in the ionic state of the amino acid residuals of the enzyme, and this will further influence the ability of substrate binding with enzyme and ultimately affect the enzymatic reaction rate of urease [63]. Calibration curves for urea biosensor between 50 mM and 500 mM urea were established at pH 6, pH 7 and pH 8. The R^2^ and sensitivity values for each response curve were calculated and presented in Table 3. The optimum biosensor response was obtained at pH 7 with the highest sensitivity compared to pH 6 and pH 8.The maximum free urease activity has also been reported at pH 7 [64]. Lower and higher pH values inhibited urease activity as the enzyme conformation is altered in both alkaline and acidic reaction media [65].

#### 3.3.5. Dynamic Linear Response Range of the Urea Biosensor

**Figure 9.**
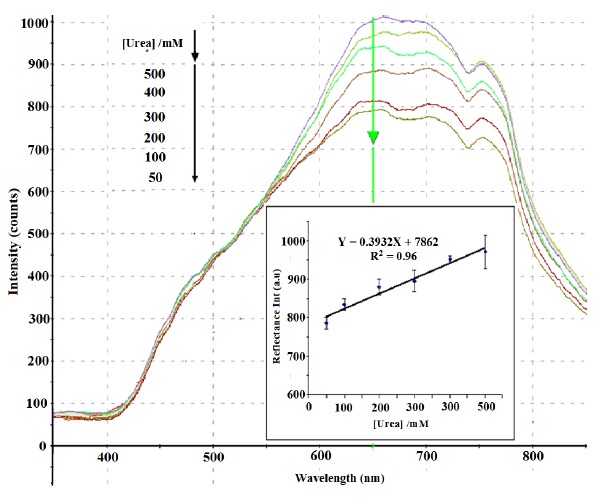
Reflectance spectra and calibration curve of urea biosensor obtained in the urea concentration range of 50–500 mM at 0.05 M phosphate buffer (pH 7) (*n* = 6).

Under optimum conditions, a linear response (Y = 0.3932X + 7862, R^2^ = 0.96) between 50 mM and 500 mM urea was obtained at the wavelength of 650 nm. The detection limit of the biosensor was 10 mM urea. At this concentration, a slight peak change was observed. The reproducibility RSD values of the biosensor was in the range of 1.7%–4.5% (*n* = 6). Figure 9 shows the reflectance spectra between 350 to 850 nm and the calibration curve of urea biosensor in 50–500 mM urea. The large response range of the SiO_2_NPs-based urea biosensor was attributed to the large surface-to-volume ratio of the SiO_2_NPs, which provided high surface reaction activity and allowed large amount of immobilized urease enzymes [66]. The high enzyme loading increased the hydrolysis of urea, and thus the linearity of the biosensor response to urea has increased to higher urea concentration [57].

#### 3.3.6. Response Time of the Biosensor

**Figure 10.**
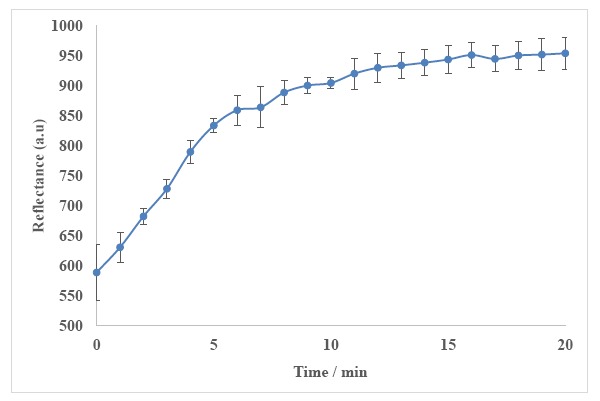
Response time of the biosensor towards 500 mM urea in 50 mM phosphate buffer at pH 7 (*n* = 3).

Response time of the biosensor is influenced by kinetic parameters, diffusion barriers, enzyme loading, enzyme activity and enzyme immobilization procedure [67]. The response time study of the biosensor was carried out for 20 min using 500 mM urea in 50 mM phosphate buffer at pH 7. When the urea solution was introduced into the biosensor surface, reflectance measurement was commenced. The reflectance reading was taken every minute until a constant reflectance response was achieved. The reflectance intensity at the wavelength of 650 nm was then plotted against time (Figure 10). Large changes in the biosensor response were observed in the first 6 min. The changes of the biosensor response became less than 4% per minute after 9 min of reaction time and remained rather constant thereafter. Thus, the response time of the optical urea biosensor is approximately 9 min.

### 3.4. SiO_2_NPs-based Optical Urea Biosensor Performance

#### 3.4.1. Recovery Performance of the Optical Urea Biosensor

**Figure 11.**
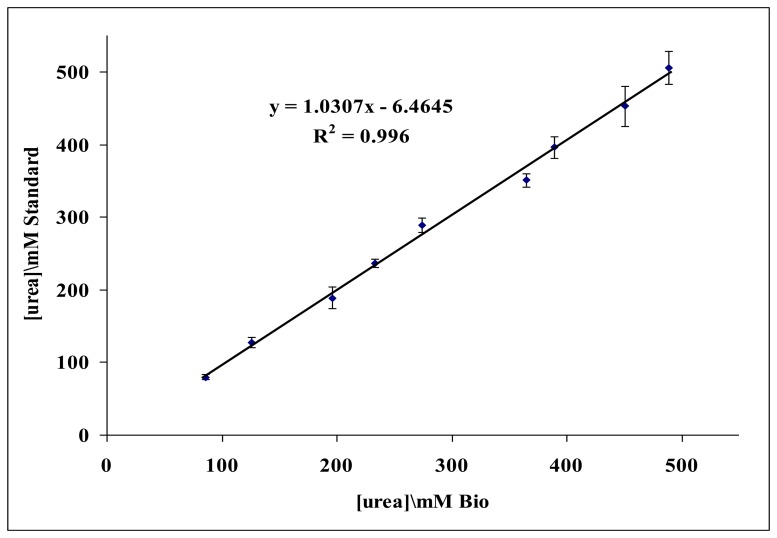
The correlation between biosensor and DMAB standard method for urea determination in urine samples (*n* = 3).

The developed reflectometric urea biosensor response was in the range of 50–500 mM, therefore this biosensor is suitable to determine urea in human urine samples as the urea concentration in human urine is reported to be in the 10–20 mg/mL (166.5–333.0 mM) range (Liu *et al.* [63]). The performance of the biosensor has been compared with the DMAB spectrophotometric method [33] for urea determination in urine samples. The working range of the DMAB method for urea determination is 0.5–7.0 mM, whereas the biosensor linear response range is from 50 to 500 mM. Therefore, dilutions of urine samples were performed to determine urea concentration using the DMAB method. Figure 11 shows the correlation between the two studied methods. The slope value obtained at 1.0307 is close to one, and thus the biosensor method is comparable to the DMAB standard method. The R^2^ value of 0.996 indicates a strong correlation between the two methods.

#### 3.4.2. Stability Study of the Biosensor

**Figure 12.**
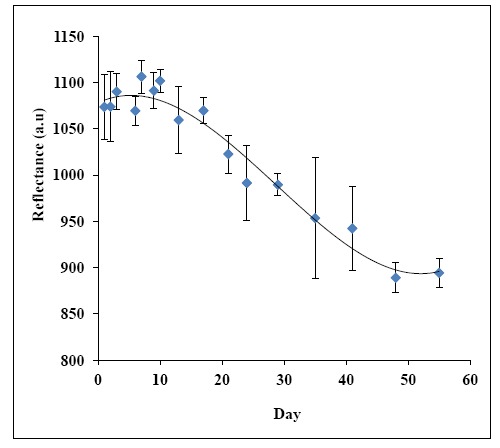
The optical urea biosensor response over 55 days experimental period using 600 mM urea in 50 mM phosphate buffer (pH 7) (*n* = 3).

Enzymes are known for their instability if they are left for a long period of time and are sensitive to their environmental conditions. Generally, the biosensor stability is governed by the stability of the sensing element e.g., the immobilized enzyme [68]. Operational life of a biosensor is important for practical applications and is governed by the immobilization method. In the present study, the urea biosensor stability was studied for about 55 days. The changes of biosensor response over 55 days towards a fixed amount of urea concentration at 500 mM in 50 mM phosphate buffer (pH 7) is shown in Figure 12. The biosensor response was found constant at high reflectance intensity for the first 17 days. Then, the biosensor response decreased gradually with time until the 48^th^ day of the experimental period, and the biosensor response maintained at low reflectance intensity thereafter. The loss of enzyme activity may be occurred in the immobilized state due to denaturation and deactivation of the enzyme, which diminished the biosensor service life [69–71]. Thus, the optical urea biosensor operational period is about 17 days.

#### 3.4.3. Selectivity of the Biosensor towards Urea

**Figure 13.**
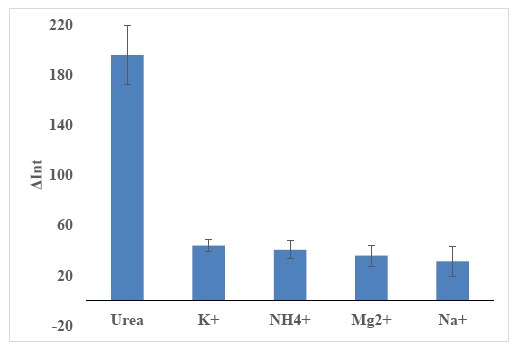
The maximum response changes of the biosensor towards various cations from 10^−5^ to 10^−1^ M and urea at the wavelength of 650 nm (*n* = 3).

The optimized urea biosensor response is expected to be selective towards urea due to the following reasons: (1) Urease is an enzyme specific to urea catalysis; (2) The chromoionophore is a pH dye sensitive towards pH changes [72]. The immobilized chromoionophore is blue in colour in protonated state and red/pink in deprotonated state [16,17], which corresponds to a reflectance wavelength of 650 nm. To confirm the biosensor selectivity, an interference study was carried out using different concentrations (10^−5^–10^−1^ M) of Na^+^, K^+^, Mg^2+^ and NH_4_^+^ ions. These cations were chosen because these cations are a common cationic species existing in our urine real sample (Olsauskaite *et al.* [73]). Based on Figure 13, it is clearly seen that the largest relative reflectance was obtained for urea, and interfering ions (*i.e.*, Na^+^, K^+^, Mg^2+^ and NH_4_^+^ ions) gave negligible responses. Therefore, it can be deduced that the biosensor is selective only towards urea.

### 3.5. Comparison with Other Nanoparticles-Based Optical Urea Biosensors

Several optical urea biosensors based on different kinds of nanoparticle immobilization matrices were reported in the literature (Table 4).

A potentiometric urea biosensor based on urease (Urs) covalently immobilized on multi-walled carbon nanotubes (MWCNTs) embedded in silica matrix deposited on the surface of an indium tin oxide (ITO) coated glass plate was fabricated by Ahuja *et al.* [49]. The biosensor showed a response in the range of 0.02–1070 mM urea with a response time of around 25 s. A reflectometric urea biosensor was fabricated by Ulianas *et al.* [74], based on succinimide-modified acrylic microspheres (nBA MPs) immobilized with a Nile blue chromoionophore and urease. The biosensor showed a response in the range of 0.01 to 1000 mM urea with a limit of detection of 9.97 μM.

The optical urea biosensor developed in this research exhibited the largest linear response range of 50–500 mM and detection limit of 10 mM urea, whilst other reported urea biosensors showed linear response range limits of up to 10 mM urea. The large surface area of SiO_2_NPs enhanced the enzyme and dye loading on the nanoparticles' surfaces. As a result, higher enzyme activity can be achieved and this yielded a larger dynamic linear concentration range of the biosensor. The silica-gel nanoparticles reduced the diffusion barriers to both reactant and product of a biochemical reaction, and shorter biosensor response times were obtained compared to microparticle-based biosensors.

Another significance of using silica-gel nanoparticles is the ability of the immobilization of both sensing materials onto the surface of silica-gel nanoparticles without a leaching problem. The weakness of this biosensor is the low detection limit. As reported by Lee *et al.* [75], Sheldon *et al.* [76] and Hanefeld *et al.* [77], ionic immobilization is a strong enzyme immobilization method but the charge of the carrier could have an effect on the residual enzyme charges or sometimes on the enzyme active site that results in the reduction of the immobilized enzyme activity. Even though the enzyme immobilization method was efficient enough to prevent enzyme leaching from the biosensor, this efficiency affected the sensitivity and the stability of the biosensor due to ionic immobilization effects.

## 4. Conclusions

Different silica-gel nano-compositions were synthesized based on the Stober method. The pH changes from the immobilized urease enzyme catalysis were found to correlate to urea concentrations, which enabled detection of urea concentrations based on the colour change of the immobilized chromoionophore dye. The use of SiO_2_NPs for urease and chromoionophore immobilizations demonstrated remarkable analytical improvement in terms of linear response range, reproducibility and selectivity. Due to the large surface area to volume ratio of SiO_2_NPs, the catalytic efficiency of the enzyme biosensor was improved as higher amounts of enzyme can be loaded. The urea biosensor has been used to determine urea concentration in urine samples, and the result was found to be in good agreement with the established DMAB method.

## Figures and Tables

**Table 1.** SiO_2_NPs chemical compositions prepared under basic medium and the average particle sizes obtained (*n* = 10).

SiO_2_NPs Composition	TEOS (mL)	EtOH (mL)	NH_3_ (mL)	H_2_O (mL)	C16TAB (mg)	Size (nm)	SD
E1	1	30	4			255.45	14.49
E7	1	24	4			285.20	14.18
E13	1	18	4			322.26	18.04
E20	1	11	4			451.10	12.03
T1	0.5	20	4			261.12	18.17
T4	0.8	20	4			285.30	10.84
T8	1.2	20	4			309.43	12.68
T11	1.5	20	4			325.72	14.77
H1	1	20	4	0.5		319.56	18.72
H5	1	20	4	2.5		292.79	14.32
H9	1	20	4	4.5		264.06	30.46
H13	1	20	4	6.5		258.22	25.02
N5	1	20	3			280.18	7.13
N9	1	20	5			316.09	19.31
N13	1	20	7			322.97	26.25
N17	1	20	9			326.76	30.04
C4	1	20	4	1.2	100	-	-
C9	1	20	4	1.2	200	-	-
C14	1	20	4	1.2	300	-	-
C19	1	20	4	1.2	400	-	-
t1	0.4	20	0.8	1.6		194.60	10.65
t2	2	20	0.8	1.6		153.94	25.04
t3	1.2	20	0.4	1.6		-	-
t4	1.2	20	1.6	1.6		263.99	13.71

E: variation in ethanol volume, T: variation in TEOS volume, H: variation in H_2_O volume, N: variation in NH_3_volume, C: variation in CTAB volume, t: trial compositions.

**Table 2.** R^2^ and sensitivity values of reflectometric urea biosensor with different amounts of chromoionophore loading (*n* = 3).

Biosensor	Chromoionophore wt/mg	Wavelength (nm)	Correlation Coefficient, R^2^	Sensitivity	Linear Range (mM)
B1	0.10	650	0.7204	0.1957	50–500
B2	0.20	650	0.9609	0.3932	50–500
B3	0.25	650	0.9384	0.3985	50–500
B4	0.64	760	0.9417	0.3056	50–500

**Table 3.** The pH effect on urea biosensor response at the wavelength of 650 nm (*n* = 3).

pH	The Correlation Coefficient, R^2^	Sensitivity	Linear Range (mM)
6	0.7896	0.2639	50–500
7	0.9609	0.3932	50–500
8	0.8637	0.2410	50–500

**Table 4.** Summary of some reported nanoparticles-based optical urea biosensors and the biosensor reported in this work.

Method	Immobilization Matrix	Linear Range (mM)	Detection Limit (mM)	Response Time (min)	Reference
UV/Vis spectrophotometric	calcium alginate microspheres	0.017–10	0.017	8	[9]
Fluorometric	Sol-gel	0–10	-	0.2	[10]
UV/Vis spectrophotometric	PANI/cellulose polyacetate membrane	1–10	1	3	[11]
potentiometric	Urs/MWCNTs/SiO2/ITO	0.0218–1070	-	0.4	[49]
Reflectance spectrophotometric	nBA MPs		0.00997	10	[74]
Reflectance spectrophotometric	SiO_2_NPs	50–500	10	6	Present work
